# Genetic Diversity From Proviral DNA as a Proxy for Time Since HIV-1 Infection

**DOI:** 10.1093/infdis/jiae149

**Published:** 2024-03-20

**Authors:** Marius Zeeb, Paul Frischknecht, Michael Huber, Corinne D Schenkel, Kathrin Neumann, Christine Leeman, Julia Notter, Andri Rauch, Marcel Stöckle, Matthias Cavassini, Enos Bernasconi, Dominique L Braun, Huldrych F Günthard, Karin J Metzner, Roger D Kouyos

**Affiliations:** Department of Infectious Diseases and Hospital Epidemiology, University Hospital Zurich, Zurich, Switzerland; Institute of Medical Virology, University Zurich, Zurich, Switzerland; Department of Infectious Diseases and Hospital Epidemiology, University Hospital Zurich, Zurich, Switzerland; Institute of Medical Virology, University Zurich, Zurich, Switzerland; Department of Infectious Diseases and Hospital Epidemiology, University Hospital Zurich, Zurich, Switzerland; Department of Infectious Diseases and Hospital Epidemiology, University Hospital Zurich, Zurich, Switzerland; Department of Infectious Diseases and Hospital Epidemiology, University Hospital Zurich, Zurich, Switzerland; Division of Infectious Diseases and Hospital Epidemiology, Cantonal Hospital St Gallen, St Gallen, Switzerland; Department of Infectious Diseases, Inselspital, Bern University Hospital, University of Bern, Bern, Switzerland; Division of Infectious Diseases and Hospital Epidemiology, University Hospital Basel, Basel, Switzerland; Medical Faculty, University of Basel, Basel, Switzerland; Division of Infectious Diseases, University Hospital Lausanne, University of Lausanne, Lausanne, Switzerland; Division of Infectious Diseases, Ente Ospedaliero Cantonale, University of Geneva and University of Southern Switzerland, Lugano, Switzerland; Department of Infectious Diseases and Hospital Epidemiology, University Hospital Zurich, Zurich, Switzerland; Department of Infectious Diseases and Hospital Epidemiology, University Hospital Zurich, Zurich, Switzerland; Institute of Medical Virology, University Zurich, Zurich, Switzerland; Department of Infectious Diseases and Hospital Epidemiology, University Hospital Zurich, Zurich, Switzerland; Institute of Medical Virology, University Zurich, Zurich, Switzerland; Department of Infectious Diseases and Hospital Epidemiology, University Hospital Zurich, Zurich, Switzerland; Institute of Medical Virology, University Zurich, Zurich, Switzerland

**Keywords:** HIV-1, next-generation sequencing, proviral diversity, infection recency, time since infection

## Abstract

HIV-1 RNA genetic diversity predicts time since infection, which is important for clinical care and research. It is unclear, however, whether proviral DNA genetic diversity sampled under suppressive antiretroviral therapy can be used for this purpose. We tested whether proviral genetic diversity from next-generation sequencing predicts time since infection and recency in 221 people with HIV-1 with known infection time. Proviral diversity was significantly associated with time since infection (*P* < 5×10^−7^, *R*^2^ up to 25%) and predictive of treatment initiation during recent infection (area under the curve-receiver operating characteristic up to 0.85). This shows the utility of proviral genetic diversity as a proxy for time since infection.

## Teaser

Knowing the time since infection with HIV is important for clinical care and research, yet it is often unknown. We show here that this time can be estimated from the diversity observed in proviral DNA.

Knowing the time since infection in people with human immunodeficiency virus type I (PWH) is relevant for transmission epidemiology, HIV therapy, and for many research questions in general. Because a longer time of infection without therapy means a longer period of ongoing replication and therefore increased viral evolution, it directly impacts the within-host viral diversity and proviral reservoir size. This has implications, for example, when deciding on simplifying antiretroviral therapy [1] or in investigations about immune responses [2]. However, its estimation is often challenging due to lack of a previous negative HIV test or recall of unambiguous risk situations leading to an infection.

As HIV diversity increases with infection time, different diversity-based approaches have been developed for estimating time since infection and especially if a PWH was recently (ie, less than 1 year) infected. For example, Kouyos et al [3] used ambiguous nucleotide frequency from Sanger sequences from routine HIV drug resistance testing, and Carlisle et al and Puller et al [4, 5] showed that an average pairwise diversity score (APD) based on next-generation sequencing (NGS) provides an even more accurate measure. In plasma virus-derived sequences from antiretroviral therapy (ART)-naive PWH, APD score correlates well with time since infection and has a receiver operating characteristic (ROC) area under the curve (AUC) of over 95% to determine if PWH were infected recently [4].

For a large number of PWH, the pre-ART sequences required for these approaches are not available. However, increasing numbers of PWH may have proviral DNA sequences performed for research purposes, or to guide treatment simplifications or treatment with long-acting antiretrovirals [6]. Such proviral DNA sequences might in principle inform the time between infection and therapy initiation, as it is expected that the diversity of the viral reservoir increases with the length of this time window, but then stops after ART has suppressed viral replication [7, 8]. However, proviral diversity also differs in important ways from pre-ART viral diversity: proviral diversity represents the accumulated diversity over the entire infection, it may be affected by the decay of the reservoir, and by hypermutations in proviral DNA caused by APOBEC3G/F [9].

As these differences may affect the association with prediction of infection time, we evaluate in this study the utility of proviral sequences sampled post-ART as a proxy for the time between infection and ART. Given the role of APOBEC3G/F as a source of noise, we combine this approach with a hypermutation filtering on a NGS read level.

## METHODS

### PWH and Sequence Selection Criteria

We included PWH with an accurate date of infection enrolled in the Swiss HIV Cohort Study (SHCS), a prospective, multicenter cohort study enrolling PWH in Switzerland [10], and/or in the Zurich Primary Infection Cohort (ZPHI) a multicenter cohort study enrolling PWH during primary HIV infection [11]. These include PWH with a negative HIV-1 test within 1 year prior to the date of diagnosis and PWH with a clinical diagnosis of a documented primary HIV infection based on a comprehensive clinical assessment by a highly experienced research team. We determined the date of diagnosis as the earliest date of the following events: SHCS registration, first HIV-1 positive test, or first HIV-1 laboratory measurement. The date of infection was defined as described previously [3]: (1) for PWH in the ZPHI as the estimated date of infection, (2) for PWH with primary infections as the date of diagnosis minus 30 days (to account for incubation time), and (3) for all others as the midpoint between diagnosis date and last negative test. We selected proviral NGS sequences from those selected PWH without ART interruption and virological failure until the day of sampling. Samples were predominantly sequenced in a study that systematically sequenced the proviral DNA of all SHCS participants without HIV-RNA genotyping available [12]. We considered the length of 2 time windows for the analysis, the number of years from the date of infection until date of ART start (t_InfectionToART_), that is, time since infection, and the time number of years from ART start until proviral NGS sequence sampling (t_ARTtoSampling_).

### NGS Sequencing

DNA was isolated from on average 5 million peripheral blood mononuclear cells and proviral DNA was amplified by (1) near full-length polymerase chain reaction (PCR) and followed by 2 nested hemilength PCRs [12]; (2) if unsuccessful, near full-length PCR followed by nested near full-length PCR; or (3) 2 hemilength PCRs amplifying a 5′ amplicon and a 3′ amplicon followed by nested hemilength PCRs was performed as previously described [7]. NGS sequencing was performed for the near full-length HIV-1 genome using the MiSeq Reagent Kit version 2 (300-cycles). Majority consensus alignments were created from the NGS reads using SmaltAlign (https://github.com/medvir/SmaltAlign). From majority consensus sequences, respective genes (*gag*, *pol, env*) were extracted with BLAST and codon alignments were made with the HIV-1 reference strain HXB2 using MACSE2 [13].

### APOBEC Hypermutation Filtering

Hypermutation filtering was performed based on a previously published method [9, 14]. We adapted this method to the level of single NGS reads, using 3 different *P* value thresholds to determine hypermutation status of a read and subsequent removal: (1) a constant threshold of *P* < .05; (2) a liberal dynamic threshold based on the bootstrapped upper 95% confidence interval (CI) of the mean from the hypermutation *P* value distribution of RNA sequences, randomly selected from the SHCS NGS database at the University Hospital Zurich, for each HIV-1 genome position (HXB2 as reference); and (3) a conservative dynamic threshold based on the bootstrapped upper 95% CI of the upper 90% percentile interval of the *P* value distribution of RNA sequences for each HIV-1 genome position (HXB2 as reference). Filters and their effect are shown as an example in Supplementary Figure 1. After filtering, we generated a new fastq file, reran SmaltAlign, and recalculated the APD.

### Average Pairwise Diversity Score

We calculated the APD score as described by Carlisle et al and Puller et al [4, 5] based on the third codon position of *gag*, *pol*, and *env* individually on the NGS sequence reads and after applying the 3 different hypermutation filters described above with a coverage threshold of 100 reads for each position.

### Time From Infection to ART and Recent Infection Analysis

We used linear regression models to determine the fraction (*R*^2^) of the variance of t_InfectionToART_ (time since infection) explained by the APD score calculated on *gag*, *pol*, and *env*. We used ROC curve analysis to determine the validity of the APD for the prediction of recent infection by the APD score calculated on *gag*, *env*, and *pol* separately and in combination for all different hypermutation thresholds. We used 2 approaches: (1) including all NGS data sets comprising at least 100 codons of the respective gene *env*/*gag*/*pol* (designated as partial length); and (2) full length, including only NGS data sets covering nearly the entire gene, that is, > 95% of codons of the respective gene *gag*/*pol/env* (designated as full length).

## RESULTS

**Figure 1.**
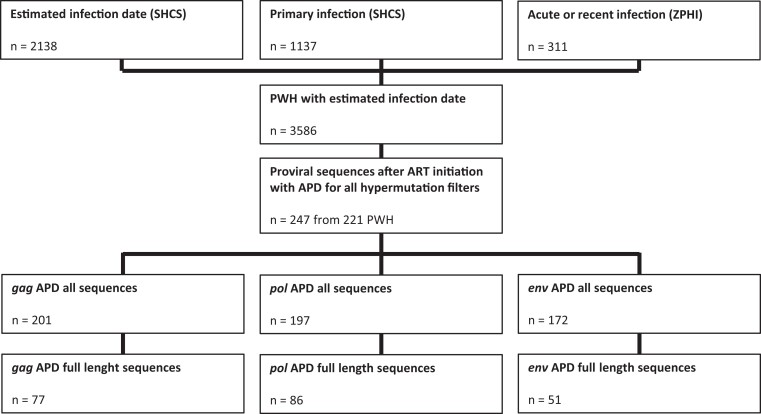
Flowchart of PWH selection and availability of HIV-1 genome sequences. Abbreviations: APD, average pairwise diversity score; ART, antiretroviral therapy; PWH, people with HIV-1; SHCS, Swiss HIV Cohort Study; ZPHI, Zurich Primary HIV Infection study.

We identified 221 PWH with a total of 247 sequences in the SHCS and ZPHI study with an accurate HIV-1 infection date reported and HIV-1 DNA NGS sequences availability. The full-length sequence was available for at least 1 of the 3 genes in 127 PWH (Figure 1). The median t_InfectionToART_ was 0.41 years (interquartile range [IQR], 0.15–2.27) and the median t_ARTtoSampling_ was 2.29 years (IQR, 0.95–4.46) (Supplementary Table 1). We also found an increasing CD4 T-cell count from 431 cells/μL (IQR, 300–627) at ART initiation to 636 cells/μL (IQR, 505–852) at the NGS sample date, and a respective decrease for HIV RNA viral load from 18 000 copies/mL (IQR, 26–146 801) to undetectable (IQR, 0–0).

**Figure 2.**
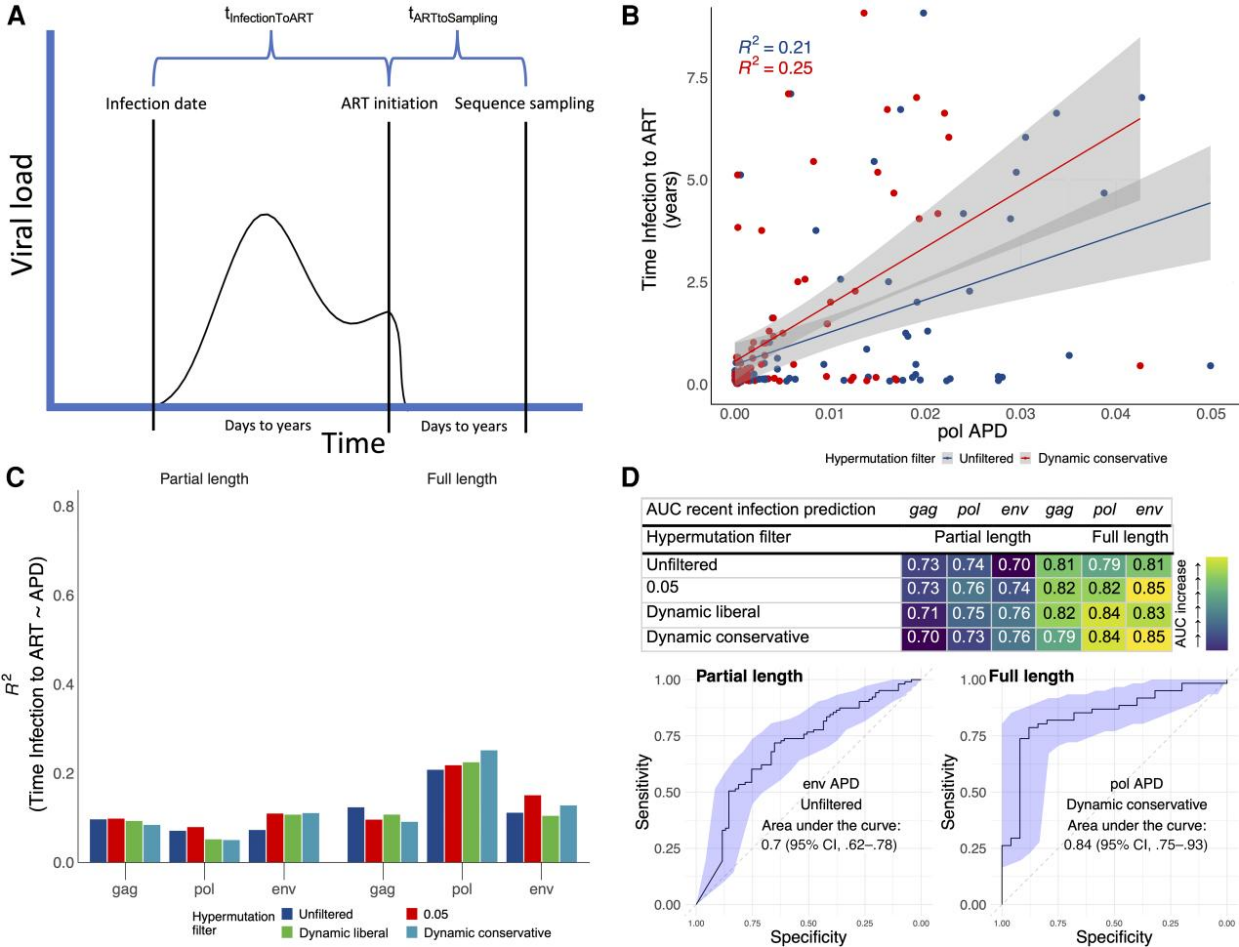
Time from HIV-1 infection to ART initiation prediction with proviral genetic diversity from NGS sequencing. *A*, Illustration of the HIV infection course and definitions of time from infection to ART initiation and time from ART initiation to proviral NGS sampling. *B*, Time of infection to ART in dependence of APD derived from full-length pol sequences. *C*, *R*^2^, the goodness of fit calculated as the explained variation in time of infection to ART by APD, of linear regression from time of infection to ART in dependence of APD derived from partial-length and restricted to full-length gag/pol/env sequences. *D*, AUCs and ROC curves for the prediction of time of infection to ART <1 year (recent infection status) with APDs derived from partial length and restricted to full-length env/pol/gag sequences. AUCs with 95% CIs are shown in Supplementary Table 4. All other ROC curves for other hypermutation filters and genes are shown in Supplementary Figures 7 and 8. *B*–*D*, Analyses were repeated for different levels of hypermutation filtering: (1) hypermutation unfiltered, (2) 0.05 threshold, (3) dynamic liberal threshold, and (4) dynamic conservative threshold (visualized at an example in Supplementary Figure 1). Abbreviations: APD, average pairwise diversity score; ART, antiretroviral therapy; AUC, area under the curve; CI, confidence interval; HIV-1, human immunodeficiency virus 1; NGS, next-generation sequencing; ROC, receiver operating characteristic.

We found significant associations of APD with t_InfectionToART_, but not with t_ARTtoSampling_ (Supplementary Table 2, and Supplementary Figures 2 and 3). Depending on the gene considered and the hypermutation-filtering threshold used, APD explained between 5% and 25% of the variance in t_InfectionToART_ (quantified as the *R*^2^ in a linear regression model; Figure 2*B* and 2*C*), with the best performance (*R*^2^ = 25%) obtained for *pol* full length and the dynamic conservative threshold. By contrast, APD explained only between 1% and 6% of the variance of t_ARTtoSampling,_ (Supplementary Figures 4–6). Overall, across genes, hypermutation filtering increases the *R*^2^ of t_InfectionToART_, in particular for *pol* full length and *env*. For *gag*, however, *R*^2^ is highest without any filtering (Figure 2*C*). When assessing the ability of APD to predict t_InfectionToART_ in leave-one-out cross validation, we found the lowest mean absolute error (MAE) in predicting t_InfectionToART_ by *pol* with dynamic conservative threshold and full length (MAE, 1.19 years). Whereas the MAE was highest for *env* (MAE, 2.19 years), with dynamic liberal threshold and full length (Supplementary Table 3).

When testing the ability of APD to predict whether ART was initiated in recent infection (<1 year), we obtained AUC ROC ranging from 0.7 (95% CI, .62–.78) for *env* without hypermutation filtering and partial length to 0.85 (95% CI, .73–.96) for *env* 0.05 and dynamic conservative threshold and full length. We found improvements of AUCs with stricter hypermutation filtering thresholds (Figure 2*C*, and Supplementary Figures 7 and 8). For *gag* APD the AUC peak was reached with the 0.05 and dynamic liberal threshold (0.82; 95% CI, .72–.92) whereas for *pol* and *env* APD the AUC peak was reached with the conservative dynamic (and 0.05) threshold, 0.84 (95% CI, .75–.93) and 0.85 (95% CI, .73–.96), respectively (Figure 2*D*).

## DISCUSSION

In this work we showed that a diversity score derived from proviral DNA HIV-1 NGS sequences from individuals on suppressive ART is associated with the time since infection (t_InfectionToART_) and recent infection status. Its predictive accuracy is lower than that of viral diversity derived from plasma HIV-1 RNA [4], in particular when partial sequences were included. However, when restricting the analysis to full-length sequences and hypermutation filtering, predictive performances are in the range of what is achieved with treatment-naive plasma RNA for *pol*/*env* (AUC of 0.84/0.85 for proviral DNA compared to ≥0.95 for viral RNA). For *gag,* hypermutation filtering showed no improvements, which may be explained by the lower G→A substitution rates in gag [15]. The performance increase comparing partial *pol* to the entire *pol* gene is striking (Figure 2*C*). This may be explained by absence of the *pol* positions 3000 to 4000 in almost 50% of sequences (Supplementary Figure 9), which previously were shown to have the highest predictability for time since infection [5]. Finally, we show that the APD only has minor associations with t_ARTtoSampling_, confirming our assumption and previous evidence [7] that there is almost no viral evolution under suppressive ART.

The main limitation of this work is the small number of recovered gene sequences, which is most likely due to low reservoir sizes in early treated PWH [1]. It may also be because of the low specificity from the hypermutation filtering and subsequent failure of NGS assembly due to a lack of reads. Another limitation is the between-sequence overlap in partial length sequences, which may impact comparability of APDs inferred from different regions within a gene. Further, we could not identify an overall optimal hypermutation filtering threshold across all genes. Nevertheless, we show improvements of both the explained variance and AUC with hypermutation filtering compared to not filtering at all.

In summary, this work shows the utility of APDs derived from proviral sequences as a proxy for the time since infection and for prediction of infection recency. This may be useful for PWH without a baseline drug resistance test to decide on treatment simplification strategies in clinical practice or to determine infection recency in HIV research, for example, to retrospectively estimate HIV-1 incidence.

## Supplementary Data

Supplementary materials are available at *The Journal of Infectious Diseases* online (http://jid.oxfordjournals.org/). Supplementary materials consist of data provided by the author that are published to benefit the reader. The posted materials are not copyedited. The contents of all supplementary data are the sole responsibility of the authors. Questions or messages regarding errors should be addressed to the author.

## Supplementary Material

jiae149_Supplementary_Data

## Notes

***Author contributions.*** M. Z., H. F. G., K. J. M., and R. D. K. contributed conceptualization. M. Z., P. F., M. H., K. N., C. R., J. N., A. R., M. S., M. C., E. B., D. L. B., H. F. G., K. J. M., and R. D. K. performed data curation. M. Z. performed formal analysis and visualization. R. D. K. and H. F. G. acquired funding. M. Z. and R. D. K. performed investigation. M. Z., C. D. S., and R. D. K. contributed methodology. R. D. K. performed project administration. H. F. G., K. J. M., and R. D. K. contributed supervision. R. D. K. performed validation. M. Z., H. F. G., K. J. M., and R. D. K. wrote the original draft. All authors contributed resources, and reviewed and edited the manuscript.

***Acknowledgments.*** The authors thank the patients who participated in the Swiss HIV Cohort Study; the physicians and study nurses for the excellent patient care provided to participants; Jan Meier, Yves Schäfer, and Océane Follonier from the Swiss HIV Cohort Study data center for data management; and Danièle Perraudin and Marianne Amstad for administration. We also thank Alexandra Calmy from the University Hospital Geneva.

***Financial support*.** This study has been financed within the framework of the Swiss HIV Cohort Study, supported by the Swiss National Science Foundation (grant number 201369) and by the Swiss HIV Cohort Study research foundation. The data are gathered by the Five Swiss University Hospitals, two Cantonal Hospitals, 15 affiliated hospitals and 36 private physicians (listed in http://www.shcs.ch/180-health-care-providers). Furthermore, this work was supported by the Swiss National Science Foundation (grant number 179571 to H. F. G.); the Yvonne-Jacob Foundation (to H. F. G.) (https://stiftungen.stiftungschweiz.ch/organisation/stiftung-yvonne-jacob); and the University of Zurich Clinical Research Priority Program Viral Infectious Diseases, Zurich Primary HIV Infection Study (to H. F. G. and D. L. B.). R. D. K. was supported by the Swiss National Science Foundation (grant numbers 324730_207957 and BSSGI0_155851).

***Data availability.*** The individual-level datasets generated or analyzed during the current study do not fulfill the requirements for open data access: (1) the SHCS informed consent states that sharing data outside the SHCS network is only permitted for specific studies on HIV infection and its complications, and to researchers who have signed an agreement detailing the use of the data and biological samples; and (2) the data is too dense and comprehensive to preserve patient privacy in persons with HIV. According to the Swiss law, data cannot be shared if data subjects have not agreed or data is too sensitive to share. Investigators with a request for selected data should send a proposal to the respective SHCS address (www.shcs.ch/contact). The provision of data will be considered by the Scientific Board of the SHCS and the study team and is subject to Swiss legal and ethical regulations, and is outlined in a material and data transfer agreement.

Presented in part: Conference on Retroviruses and Opportunistic Infections (CROI), Denver, 3–6 March, 2024.
