# Supplementary material for: Genetic Diversity From Proviral DNA as a Proxy for Time Since HIV-1 Infection
Source: J Infect Dis. 2024 Mar 20;230(3):e631–6. doi: 10.1093/infdis/jiae149 (PMC11420808; doi:10.1093/infdis/jiae149)
Supplement: jiae149_Supplementary_Data [file jiae149_supplementary_data.docx]

**Supplementary Material**

Supplementary Table 1 Characteristics of study population

| **Characteristic** | **N = 221***^1^* |  |
| --- | --- | --- |
| Sex |  |  |
| Female | 28 (13%) |  |
| Ethnicity |  |  |
| White | 190 (86%) |  |
| Black | 14 (6.3%) |  |
| Hispano-American | 5 (2.3%) |  |
| Asian | 9 (4.1%) |  |
| Other | 3 (1.4%) |  |
| Likely HIV transmission source |  |  |
| MSM | 150 (68%) |  |
| HET | 48 (22%) |  |
| IDU | 12 (5.4%) |  |
| Other | 11 (5.0%) |  |
| Age at ART initiation (years) | 35 (29, 42) |  |
| Age at NGS sampling (years) | 38 (32, 45) |  |
| HIV-1 subtype B | 158 (71%) |  |
| CD4 at ART initiation (cells/μl) | 431 (300, 627) |  |
| CD4 at NGS sampling (cells/μl) | 636 (505, 852) |  |
| RNA at ART initiation (copies/ml) | 18,000 (26, 146,801) |  |
| RNA at NGS sampling (copies/ml) | 0 (0, 0) |  |
| Time Infection To ART (years) | 0.41 (0.15, 2.27) |  |
| Time ART To Sample (years) | 2.29 (0.95, 4.46) |  |
| Partial *gag* sequence length (number of codons) | 306 (188, 420) |  |
| Partial *pol* sequence length (number of codons) | 499 (337, 716) |  |
| Partial *env* sequence length (number of codons) | 432 (314, 594) |  |
| *^1^*n (%); Median (IQR) | | |

Supplementary Table 2 APD associations (shown as Pvalues) with t_InfectionToART_ and t_ARTtoSampling_ stratified by sequence quality, gene, and hypermutation filtering.

| Gene | Hypermutation filter | t_InfectionToART_ | t_ARTtoSampling_ | t_InfectionToART_ | t_ARTtoSampling_ |
| --- | --- | --- | --- | --- | --- |
|  |  | Partial length | | Full length | |
| *gag* | Unfiltered | 4.79e-06 | 0.186 | 1.98e-03 | 0.552 |
| *gag* | 0.05 | 4.96e-06 | 0.352 | 6.87e-03 | 0.895 |
| *gag* | Dynamic liberal | 1.12e-05 | 0.612 | 4.06e-03 | 0.961 |
| *gag* | Dynamic conservative | 3.69e-05 | 0.925 | 8.47e-03 | 0.960 |
| *pol* | Unfiltered | 1.36e-04 | 0.266 | 1.27e-05 | 0.779 |
| *pol* | 0.05 | 6.07e-05 | 0.462 | 4.37e-06 | 0.330 |
| *pol* | Dynamic liberal | 1.35e-03 | 0.557 | 2.06e-06 | 0.161 |
| *pol* | Dynamic conservative | 1.61e-03 | 0.523 | 4.72e-07 | 0.184 |
| *env* | Unfiltered | 3.60e-04 | 0.489 | 2.32e-02 | 0.111 |
| *env* | 0.05 | 9.41e-06 | 0.470 | 6.93e-03 | 0.103 |
| *env* | Dynamic liberal | 1.29e-05 | 0.661 | 2.82e-02 | 0.187 |
| *env* | Dynamic conservative | 9.97e-06 | 0.983 | 1.39e-02 | 0.159 |

Supplementary Table 3 MAEs of prediction of t_InfectionToART_ using APD with leave one out cross validation stratified by sequence quality, gene, and hypermutation filtering.

| Gene | Hypermutation filter | MAE partial length | MAE full length |
| --- | --- | --- | --- |
| *gag* | Unfiltered | 1.59 | 1.47 |
| *gag* | 0.05 | 1.59 | 1.55 |
| *gag* | Dynamic liberal | 1.59 | 1.52 |
| *gag* | Dynamic conservative | 1.62 | 1.57 |
| *pol* | Unfiltered | 1.72 | 1.26 |
| *pol* | 0.05 | 1.71 | 1.24 |
| *pol* | Dynamic liberal | 1.75 | 1.23 |
| *pol* | Dynamic conservative | 1.76 | 1.19 |
| *env* | Unfiltered | 1.98 | 2.14 |
| *env* | 0.05 | 1.88 | 2.03 |
| *env* | Dynamic liberal | 1.85 | 2.19 |
| *env* | Dynamic conservative | 1.86 | 2.14 |

Supplementary Table 4 AUCs of recent infection status prediction with APD stratified by sequence quality, gene, and hypermutation filtering.

| AUC recent infection  prediction | *gag* | | *pol* | | *env* | | *gag* | | *pol* | | *env* | |
| --- | --- | --- | --- | --- | --- | --- | --- | --- | --- | --- | --- | --- |
| Hypermutation  filter | Partial length AUC 95% CI | | | | | | Full length AUC 95% CI | | | | | |
| Unfiltered | 0.73 | 0.65-0.8 | 0.74 | 0.67-0.81 | 0.70 | 0.62-0.78 | 0.81 | 0.71-0.91 | 0.79 | 0.7-0.89 | 0.81 | 0.69-0.93 |
| 0.05 | 0.73 | 0.65-0.8 | 0.76 | 0.69-0.83 | 0.74 | 0.66-0.82 | 0.82 | 0.72-0.92 | 0.82 | 0.73-0.91 | 0.85 | 0.73-0.96 |
| Dynamic liberal | 0.71 | 0.64-0.79 | 0.75 | 0.67-0.82 | 0.76 | 0.68-0.84 | 0.82 | 0.72-0.92 | 0.84 | 0.75-0.92 | 0.83 | 0.71-0.96 |
| Dynamic conservative | 0.70 | 0.63-0.77 | 0.73 | 0.65-0.8 | 0.76 | 0.69-0.84 | 0.79 | 0.68-0.9 | 0.84 | 0.75-0.93 | 0.85 | 0.73-0.96 |


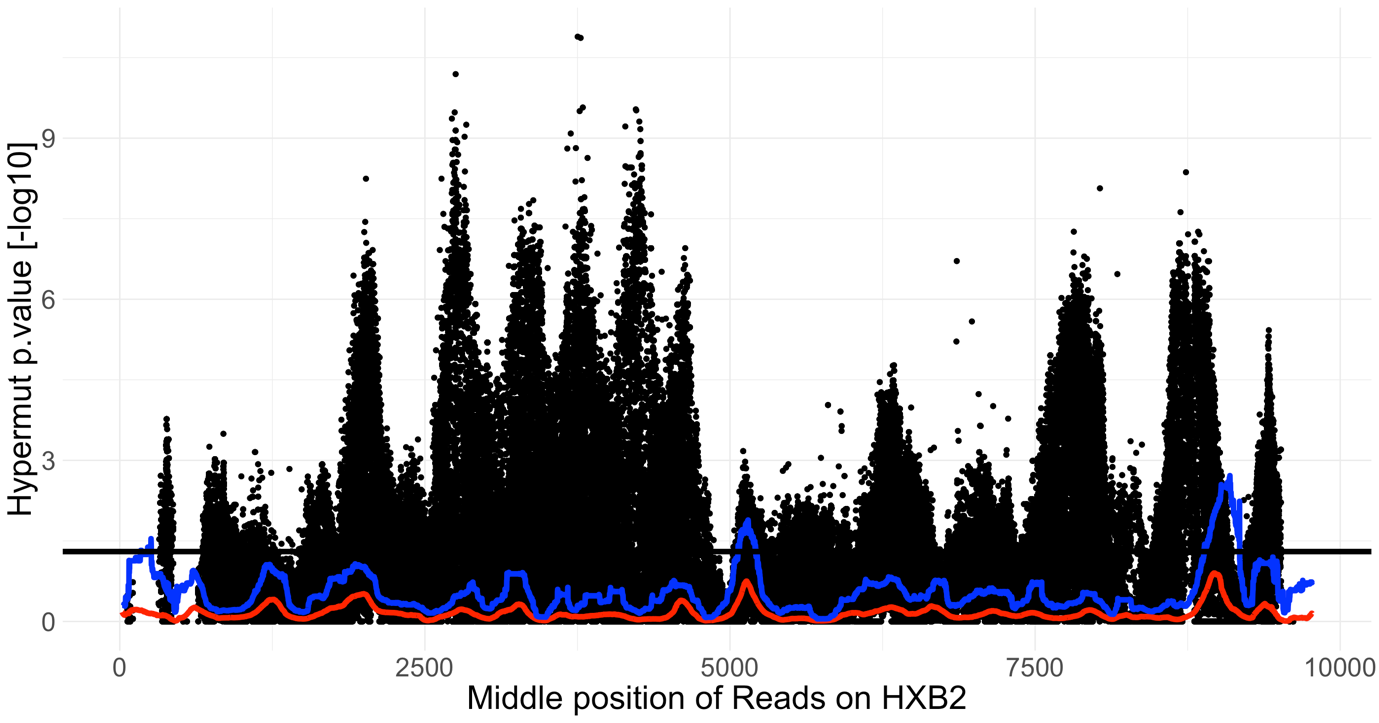


Supplementary figure 1 NGS reads, from an example sequence, shown by their position within HXB2 and their respective -log10 Pvalue of the fisher test from hypermut 2.0 (Rose & Korber, 2000). A read is hypermutated if above the thresholds. Respective, the black line is the 0.05 filter, the blue line the dynamic liberal threshold, and the red line the dynamic conservative threshold.

Supplementary figure 2 Time of infection to ART initiation in dependence of APD derived from full length *gag*/*pol*/*env* sequences (i) hypermutation unfiltered and (ii) filtered with the strictest threshold (Dynamic cons.). APD, average pairwise diversity score; ART, anti-retroviral therapy.

Supplementary figure 3 Time of infection to ART initiation in dependence of APD derived from partial length *gag*/*pol*/*env* sequences (i) hypermutation unfiltered and (ii) filtered with the strictest threshold (Dynamic cons.). APD, average pairwise diversity score; ART, anti-retroviral therapy.

Supplementary figure 4 Time of ART initiation until proviral NGS sample in dependence of APD derived from full length *gag*/*pol*/*env* sequences (i) hypermutation unfiltered and (ii) filtered with the strictest threshold (Dynamic cons.). APD, average pairwise diversity score; ART, anti-retroviral therapy.

Supplementary figure 5 Time of ART initiation until proviral NGS sample in dependence of APD derived from partial length *gag*/*pol*/*env* sequences (i) hypermutation unfiltered and (ii) filtered with the strictest threshold (Dynamic cons.). APD, average pairwise diversity score; ART, anti-retroviral therapy.

Supplementary figure 6 R^2^ of linear regression from time of ART start to sequence sample date in dependence of APD derived from partial length and restricted to full length *gag*/*pol*/*env* sequences (i) hypermutation unfiltered, (ii) 0.05 threshold, (iii) dynamic liberal threshold, and (iv) dynamic conservative threshold. APD, average pairwise diversity score; ART, anti-retroviral therapy.


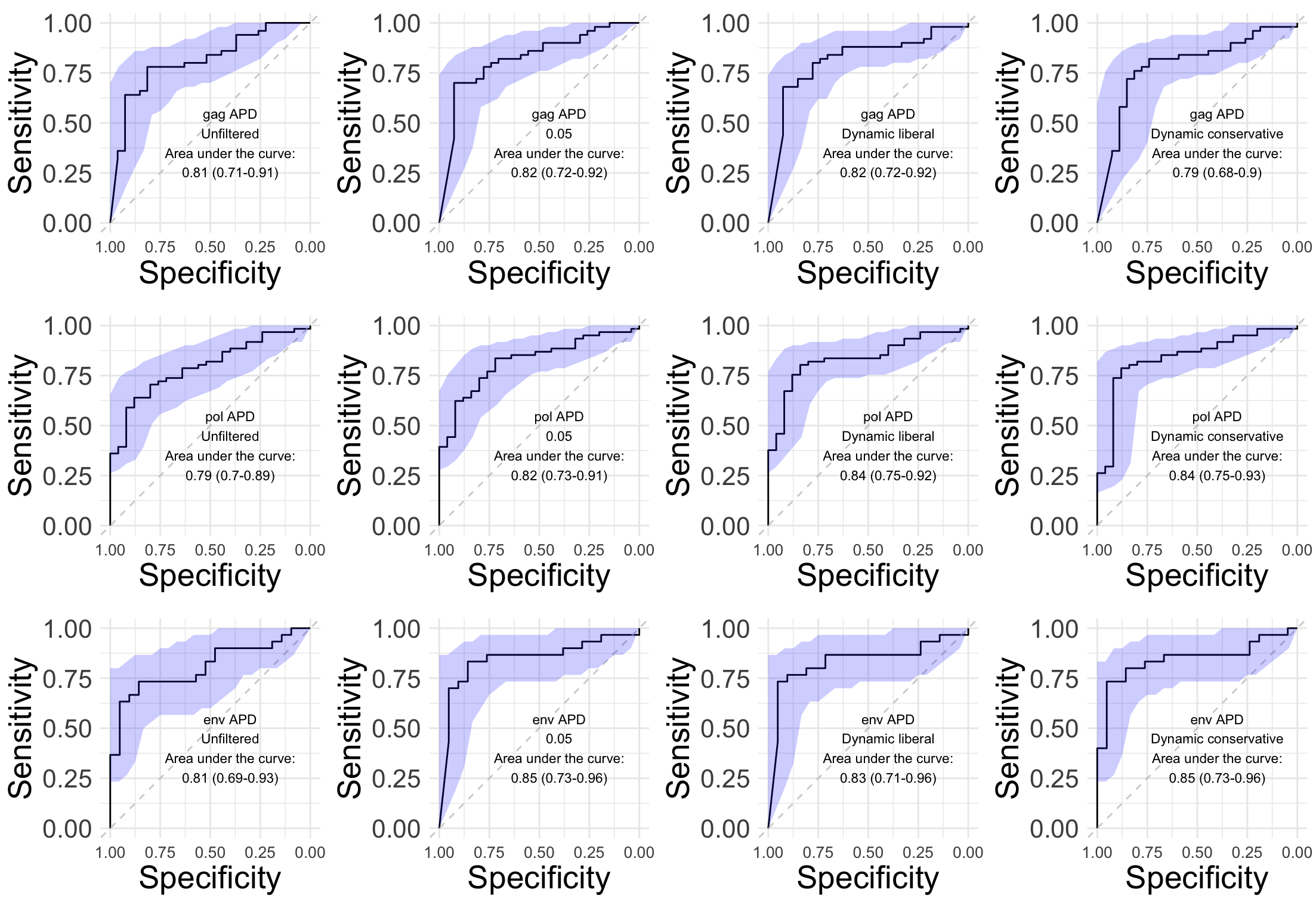


Supplementary figure 7 AUC ROC curves for the prediction of time of infection to ART start <1year (recent infection status) with APDs derived from full length *gag*/*pol*/*env* sequences (i) hypermutation unfiltered, (ii) 0.05 threshold, (iii) dynamic liberal threshold, and (iv) dynamic conservative threshold. APD, average pairwise diversity score; ART, anti-retroviral therapy; AUC, area under the curve; ROC, receiver operating characteristic.


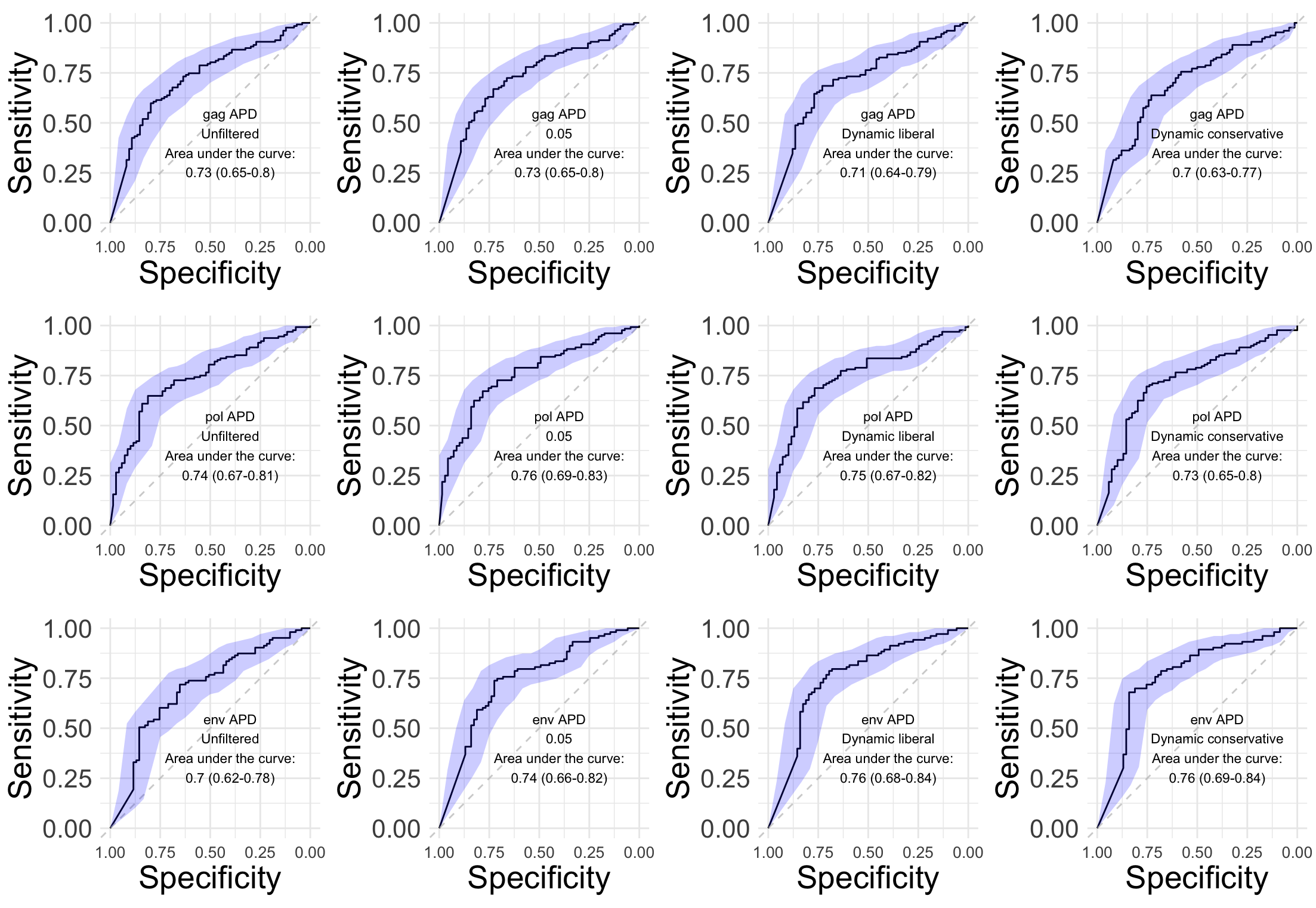


Supplementary figure 8 AUC ROC curves for the prediction of time of infection to ART start <1year (recent infection status) with APDs derived from partial length *gag*/*pol*/*env* sequences (i) hypermutation unfiltered, (ii) 0.05 threshold, (iii) dynamic liberal threshold, and (iv) dynamic conservative threshold. APD, average pairwise diversity score; ART, anti-retroviral therapy; AUC, area under the curve; ROC, receiver operating characteristic.


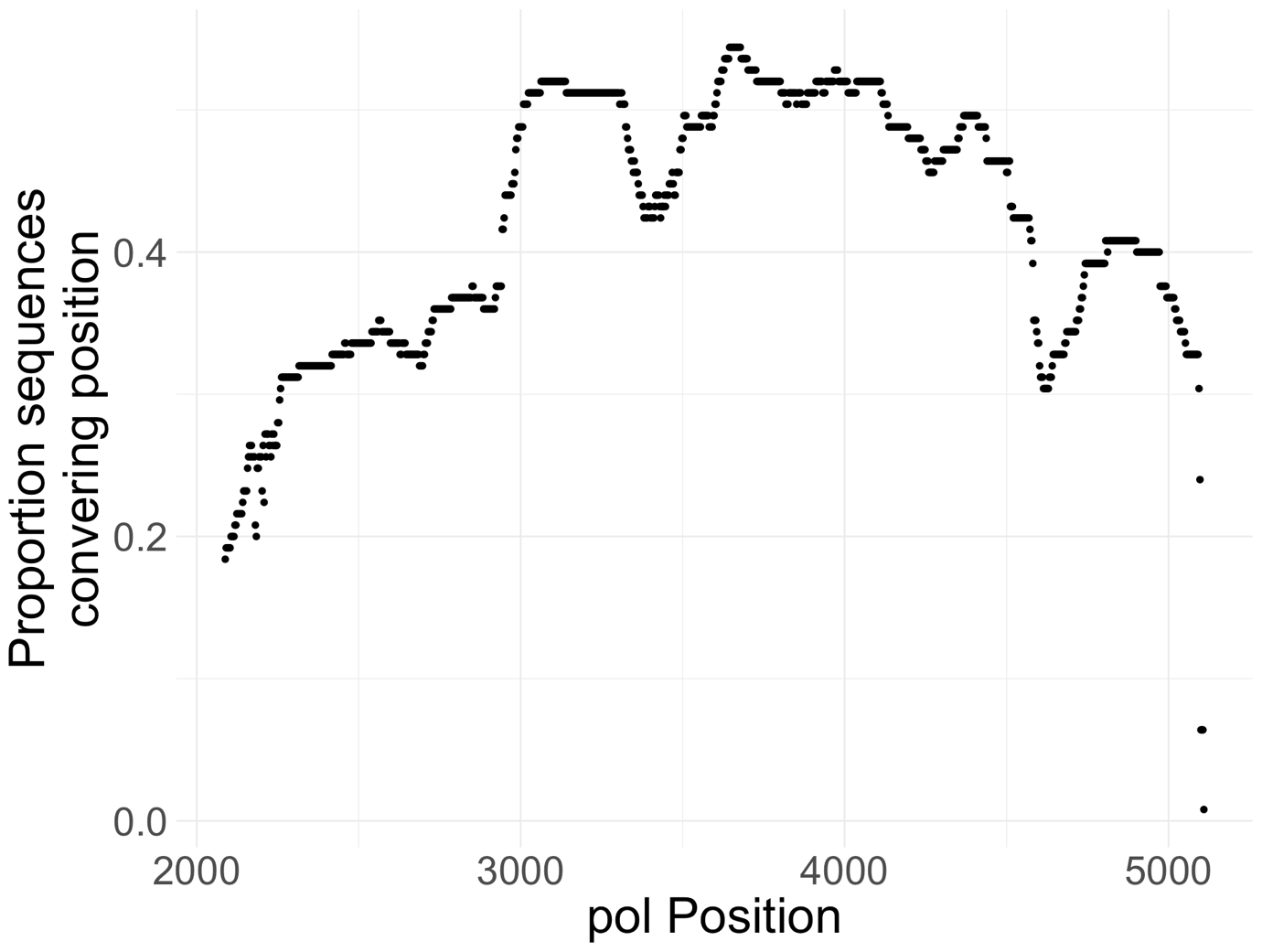


Supplementary figure 9 Partial length *pol* sequences and proportion of non-missing nucleotides per position in the multiple sequence alignment.
